# Advances in gut microbiome in metabonomics perspective: based on bibliometrics methods and visualization analysis

**DOI:** 10.3389/fcimb.2023.1196967

**Published:** 2023-05-30

**Authors:** Jieyan Wang, Peng Dong, Shuqian Zheng, Yiyin Mai, Jianan Ding, Pinfei Pan, Liugang Tang, Yantong Wan, Hui Liang

**Affiliations:** ^1^ Department of Urology, The People's Hospital of Longhua, The Affiliated Hospital of Southern Medical University, Shenzen, China; ^2^ College of Anesthesiology, Southern Medical University, Guangzhou, China; ^3^ The First School of Clinical Medicine, Southern Medical University, Guangzhou, China; ^4^ School of Basic Medical Science, Southern Medical University, Guangzhou, China; ^5^ The Second School of Clinical Medicine, Southern Medical University, Guangzhou, China; ^6^ Tendon and Injury Department, Sichuan Provincial Orthopedics Hospital, Chengdu, China; ^7^ Guangdong Provincial Key Laboratory of Proteomics, Southern Medical University, Guangzhou, China

**Keywords:** gut microbiome, metabonomics, bibliometrics, cluster analysis, visualization analysis

## Abstract

**Background and aims:**

Gastrointestinal microbial metabolomics is closely related to the state of the organism and has significant interaction with the pathogenesis of many diseases. Based on the publications in Web of Science Core Collection(WoSCC) from 2004 to 2022, this study conducted a bibliometric analysis of this field, aiming to understand its development trend and frontier, and provide basic information and potential points for in-depth exploration of this field.

**Methods:**

All articles on gastrointestinal flora and metabolism published from 2004 to 2022 were collected and identified in WoCSS. CiteSpace v.6.1 and VOSviewer v.1.6.15.0 were used to calculate bibliometric indicators, including number of publications and citations, study categories, countries/institutions, authors/co-cited authors, journals/co-cited journals, co-cited references, and keywords. A map was drawn to visualize the data based on the analysis results for a more intuitive view.

**Results:**

There were 3811 articles in WoSCC that met our criteria. Analysis results show that the number of publications and citations in this field are increasing year by year. China is the country with the highest number of publications and USA owns the highest total link strength and citations. Chinese Acad Sci rank first for the number of institutional publications and total link strength. Journal of Proteome Research has the most publications. Nicholson, Jeremy K. is one of the most important scholars in this field. The most cited reference is “Gut flora metabolism of phosphatidylcholine promotes cardiovascular disease”. Burst detection indicates that Urine, spectroscopy, metabonomic and gut microflora are long-standing hot topics in this field, while autism spectrum disorder and omics are likely to be at the forefront of research. The study of related metabolic small molecules and the application of gastrointestinal microbiome metabolomics in various diseases are currently emerging research directions and frontier in this field.

**Conclusion:**

This study is the first to make a bibliometric analysis of the studies related to gastrointestinal microbial metabolomics and reveal the development trends and current research hotspots in this field. This can contribute to the development of the field by providing relevant scholars with valuable and effective information about the current state of the field.

## 1 Introduction

The human intestinal microorganism ecosystem, called the “microbiota”, consists of bacteria, viruses, fungi, parasites, and archaea(Adak and Khan, 2019). They are responsible for important metabolic, immune and nutritional functions. The bacteria in the stomach, duodenum and jejunum are mostly aerobic Gram-positive bacteria of oropharyngeal origin, while the ileum is dominated by coliforms. After the ileocecal valve, the anaerobic bacterial species increased, mainly Bacillus spp., Bifidobacterium spp., Clostridium spp. and Lactobacillus spp(Bull and Plummer, 2014; Sidhu and van der Poorten, 2017). A normal healthy gut microbiome is highly diverse, stable and able to work with the host immune system to resist changes associated with stress(Ruan et al., 2020). Ecological dysbiosis implies a maladaptive imbalance in the Gastrointestinal microbiome and is associated with many common diseases such as inflammatory bowel diseases, bacterial infections, etc.(Gallo et al., 2016). In addition, the gastrointestinal microbiome is one of the important regulatory components of metabolic function and can provide a wealth of information on potential metabolic pathways and metabolomics. It is closely associated with common diseases such as irritable bowel syndrome, obesity, diabetes and cardiovascular diseases(Bäckhed et al., 2007; Chen and Gerszten, 2020; Erdrich et al., 2020; Jin and Ma, 2021). Therefore, the gut microbiome has become an emerging research hotspot in recent years.

Metabolomics studies can provide measurements of a large number or all metabolites in cells, tissues, organs or organisms whose changes are directly correlated with changes in biological phenotypes. Thus, metabolomics research tools based on liquid chromatography-mass spectrometry (LC-MS), gas chromatography-mass spectrometry (GC-MS), mass spectrometry (MS), nuclear magnetic resonance (NMR) and other technologies are now widely used in the diagnosis and research of various diseases, such as finding biomarkers for the early diagnosis of various diseases and studying the correlation between metabolic profiles and tumor indicators(Rinschen et al., 2019; Tseng and Wu, 2019; Wilson et al., 2021).

Gut microbiome metabolomics also plays a key role in the maintenance of organismal health and disease pathogenesis. One of the main ways in which the gut flora interacts with the host is through their metabolites. Many scholars are already exploring the microbiome of gastrointestinal microbiome and various states of the organism and related mechanisms, such as the use of fecal metabolomics to explore the interaction of disease with gastrointestinal microbiome and its metabolome(Gika et al., 2019), Use of metabolomics to analyze alterations in the gut microbiome of patients with atrial fibrillation, etc.(Chen et al., 2019). These explorations aim to identify small molecules with relevance to the association of gut microbes and their metabolites with various disease mechanisms and clinical diagnostic and therapeutic relevance, and to clarify specific regulatory mechanisms as a basis for finding additional therapeutic targets. Nevertheless, the research directions and hotspots in this field are still unclear. Thereby, we can conduct a literature analysis of the research in the field of metabolomics of the gastrointestinal microbiome to make the best use of the existing research base and to identify the research hotspots in this field.

Published bibliometric analysis reports on research hotspots, geographic distribution and temporal trends in diabetes and gastrointestinal microbiome, microbe-gut-brain axis, coronary artery disease and metabolomics, etc.(Wang et al., 2022; Yu et al., 2022; Zhang et al., 2022). However, there is still little statistical and analysis of comprehensive and detailed literature data in the field of metabolomics of gastrointestinal microbiome. Consequently, there is a need for qualitative and quantitative analysis of articles related to the metabolomics of the gastrointestinal microbiome. Bibliometrics is a widely used statistical method that uses numerical, factual, and other evaluation techniques to measure data and metrics collected in a specific field of study. The use of bibliometric and visualization software provides an objective picture of the current state of the field and provides researchers with visual information and potential research directions. Currently, bibliometrics are used in the fields of periodontology, community science, bone disease, cancer prognosis and care, and diabetes(Ahmad and Slots, 2020; Yang et al., 2021; Hadid et al., 2022; Hou et al., 2022; Zhang et al., 2022). But no articles have been published to analyze indicators of article quality, research hotspots and directions of development in metabolomics of the gastrointestinal microbiome. On this ground, the bibliometric study of this topic will complement the existing knowledge in the field, provide important information about the field and reveal the complex interrelationships between different directions of research in the field. A total of 3811 studies from 2004-2022 were included in this bibliometric analysis, presenting basic research and future trends in the metabolomics of the gastrointestinal microbiome, and providing more detailed data and clear directions for further exploration by researchers.

## 2 Materials and methods

### 2.1 Data collection

Science Citation Index Expanded of the Web of Science Core Collection (WoSCC) is an online database containing standardized and up-to-date reference data sets for scientific research and analysis. We use the search formula ((TS=(Metabolomics OR Metabolomic OR Metabonomics OR Metabonomic) AND TS=(“Gastrointestinal Microbiome” OR “Gastrointestinal Microbiomes” OR”Microbiome, Gastrointestinal” OR”Gut Microbiome” OR “Gut Microbiomes” OR”Microbiome, Gut” OR “Gut Microflora” OR “Microflora, Gut” OR “Gut Microbiota” OR “Gut Microbiotas” OR “Microbiota, Gut” OR “Gastrointestinal Flora” OR “Flora, Gastrointestinal” OR “Gut Flora” OR “Flora, Gut” OR “Gastrointestinal Microbiota” OR “Gastrointestinal Microbiotas” OR “Microbiota, Gastrointestinal” OR “Gastrointestinal Microbial Community” OR “Gastrointestinal Microbial Communities” OR “Microbial Community, Gastrointestinal” OR “Gastrointestinal Microflora” OR “Microflora, Gastrointestinal” OR “Gastric Microbiome” OR “Gastric Microbiomes” OR “Microbiome, Gastric” OR “Intestinal Microbiome” OR “Intestinal Microbiomes” OR “Microbiome, Intestinal” OR “Intestinal Microbiota” OR “Intestinal Microbiotas” OR “Microbiota, Intestinal” OR “Intestinal Microflora” OR “Microflora, Intestinal” OR “Intestinal Flora” OR “Flora, Intestinal” OR “Enteric Bacteria” OR “Bacteria, Enteric”))to search. In total, we retrieved 3811 original English-language documents, including articles and reviews, related to gastrointestinal microbiome and metabolomics from January 1, 2004 to December 29, 2022. The bibliometric results were also visualized using image format.

### 2.2 Data analysis

We counted the publications, document types, countries/regions, institutions, authors, journals, references, and keywords in the retrieved literature, removed all spelling errors, and merged identical author names and synonymous keywords. The processed data were then imported into CiteSpace v.6.1 and VOSviewer v.1.6.15.0 for bibliometric and visual analysis. CiteSpace is a Java application for bibliometric analysis. It is an interactive analysis tool that enables visual analysis through a combination of bibliometrics, visual analysis methods and data mining algorithms (Chen, 2006). VOSviewer is mainly used for bibliometric network diagram analysis (van Eck and Waltman, 2010). We used both software to analyze the distribution of countries/regions in the field of gastrointestinal microbiome metabolomics, the collaboration of various institutions, authors and their collaborations, and the regional distribution of topics. In addition, we analyzed the reference collaboration, reference burst, keywords burst, keyword timeline graph, and publication and citation trends of the literature in the field. This information can help us determine the overall academic research scope and trends in the field, clarify the basic research data and the content and evolution process in the field, and thus infer the research focus of the next field development and identify the frontier hotspots.

## 3 Results

### 3.1 General trend

**Figure 1:**
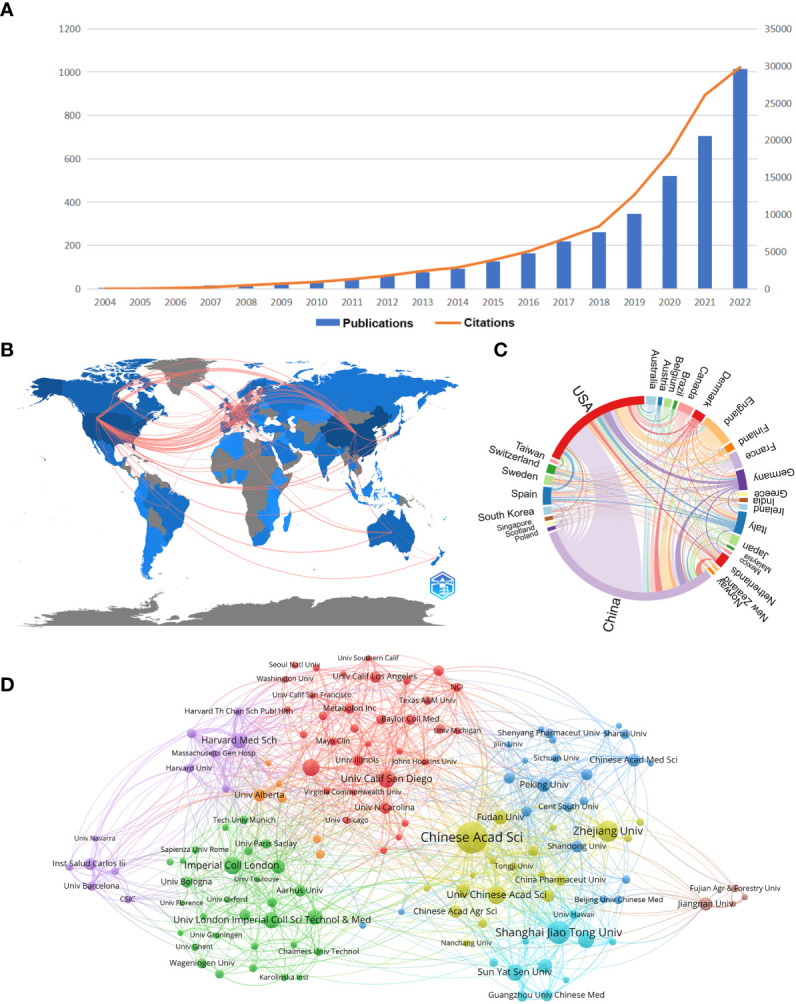
**(A)** Trends in the literature related to metabolomics of gastrointestinal flora over the past nineteen years. **(B)** Country collaboration map in the field of metabolomics of gastrointestinal microbiome. Lines represent the collaboration of the countries. **(C)** Country/region co-occurrence map of metabolomics studies on the microbiome of gastrointestinal bacteria. The size of the percentage of circles is proportional to the number of articles published in that country/region, and the thickness of the connection reflects the strength of the partnership. **(D)** Gastrointestinal microbiome metabonomics relevant institutions cluster analysis. Each node represents an institution, and the size of the circle is proportional to the number of articles published by that institution. The higher the centrality of a node, the more times it appears in the shortest path of the whole network, and the greater its influence and significance. Node connections show correlation strength, and more connections indicate more cooperation. (From: VOSviewer, CiteSpace doi: 10.3389/fcimb.2023.1196967).

The distribution of documents and citations for each period indicates the general examination patterns of the field. **Figure 1A** shows the annual publications and annual citation frequency of relevant articles from 2004 to 2022. Overall, there is an increasing trend of publications and citations on gastrointestinal microbiome metabolomics year by year, with 46.89% of mean growth rate in publications and 77.24% in citations. 2022 has the highest number of publications (1014) and the highest number of citations (29800). The annual citation frequency growth trend, on the other hand, began to moderate after peaking in 2021, but it could tolerated to fit well to exponential curve at 95% prediction bound with predict growth rate in 66.7%, meaning that the trend of citation whether to soar or descend should be observed (data not shown).

### 3.2 Countries/regions and institutions contribution

**Table 1** shows the top ten countries/regions in terms of number of papers and citations, which can reflect those countries/regions with the most outstanding contributions in the field. China, USA and England occupy the top three positions in the ranking of number of publications, total link strength and number of citations. In the ranking of number of publications, only China (1746) and USA (1036) have more than 1000 publications, while the third ranked England has 292 publications. In the ranking of citations, USA still ranks first with 57013, China ranks second (28092), and England ranks third (19283). In the total link strength ranking, USA ranked first (686), followed by England (454) and China (386).

**Table 1:** Top 10 countries in terms of number of publications, frequency of citations, and total association intensity.

Rank	Countries	Documents	Countries	Total Link Strength	Countries	Citations
1	China	1746	USA	686	USA	57013
2	USA	1036	England	454	China	28092
3	England	292	China	386	England	19283
4	Italy	209	Germany	306	Germany	8703
5	Germany	189	Italy	271	Italy	8346
6	Spain	163	France	262	Switzerland	7543
7	France	154	Netherlands	228	France	7415
8	Canada	135	Spain	190	Canada	7063
9	Netherlands	121	Canada	183	Netherlands	6718
10	Denmark	104	Sweden	173	Sweden	5217

As can be seen from **Figure 1B**, it is worth noting that the linkages between countries/regions are mainly concentrated between North America and Europe, and North America and East Asia. Whereas, Australia has almost equal but less connections with North America, Europe and East Asia. And the countries with more publications also cooperate more closely with each other than those with fewer publications. The cooperation relationship between countries or regions was visualized and analyzed, as shown in the chord diagram in **Figure 1C**. China has the highest number of publications, followed by the United States and the United Kingdom. The United States has the closest collaborative relationship with China, while the United States has the most extensive collaboration with other countries/regions.

**Table 2** shows the top 10 institutions in terms of number of publications and frequency of collaboration with other institutions. Five institutions in the top 10 in terms of number of publications as well as total link strength are from China. In the ranking of number of publications, Chinese Acad Sci had 166 publications, which was the most productive institution, followed by Shanghai Jiao Tong Univ (103 publications) and Zhejiang Univ (92) publications. In the total link strength rank, Chinese Acad Sci ranked first (224), followed by Harvard Med Sch (131) and Shanghai Jiao Tong Univ (127). Moreover, we conducted a cluster analysis of the published institutions, aiming to understand the global distribution of metabolomics research on gastrointestinal microbiome and to provide reference data for collaboration among institutions. **Figure 1D** divides all institutions into eight closely collaborating segments by color. We can observe that all these institutions cooperate closely in their respective clusters, but the connection between each cluster is not that frequent. The relatively large network of collaborating institutions is the red cluster, which includes Univ Calif San Diego, Univ Calif Davis, and Univ N Carolina. The yellow cluster showcases collaborations including Chinese Acad Sci, Zhejiang Univ, and Univ Chinese Acad Sci. The sky-blue cluster includes important institutions such as Shanghai Jiao Tong Univ, Sun Yat Sen Univ and Guangzhou Univ Chinese Med. The green cluster includes Imperial Coll London, Univ London Imperial Coll Sci.The purple cluster includes Harvard Med Sch, Inst Salud Carlos lii, and Harvard Univ. The dark blue cluster includes Peking Univ, Chinese Acad Med Sci, and Cent South Univ. The orange and brown clusters include a smaller number of institutions.

**Table 2:** Top 10 institutions in terms of number of articles issued and intensity of association.

Rank	Institution	Publications	Original Country	Institution	Total Link Strength	Original Country
1	Chinese Acad Sci	166	China	Chinese Acad Sci	224	China
2	Shanghai Jiao Tong Univ	103	China	Harvard Med Sch	131	USA
3	Zhejiang Univ	92	China	Shanghai Jiao Tong Univ	127	China
4	Univ Chinese Acad Sci	72	China	Univ Chinese Acad Sci	126	China
5	Imperial Coll London	68	England	Zhejiang Univ	84	China
6	Univ Calif San Diego	68	USA	Univ Calif San Diego	82	USA
7	Univ Calif Davis	66	USA	Brigham & Womens Hosp	80	USA
8	Sun Yat Sen Univ	64	China	Univ Copenhagen	76	Denmark
9	Univ London Imperial Coll Sci Technol & Med	60	England	Peking Univ	75	China
10	Shanghai Univ Tradit Chinese Med	59	China	Univ Alberta	75	Canada

### 3.3 Author and co-cited-author

**Figure 2:**
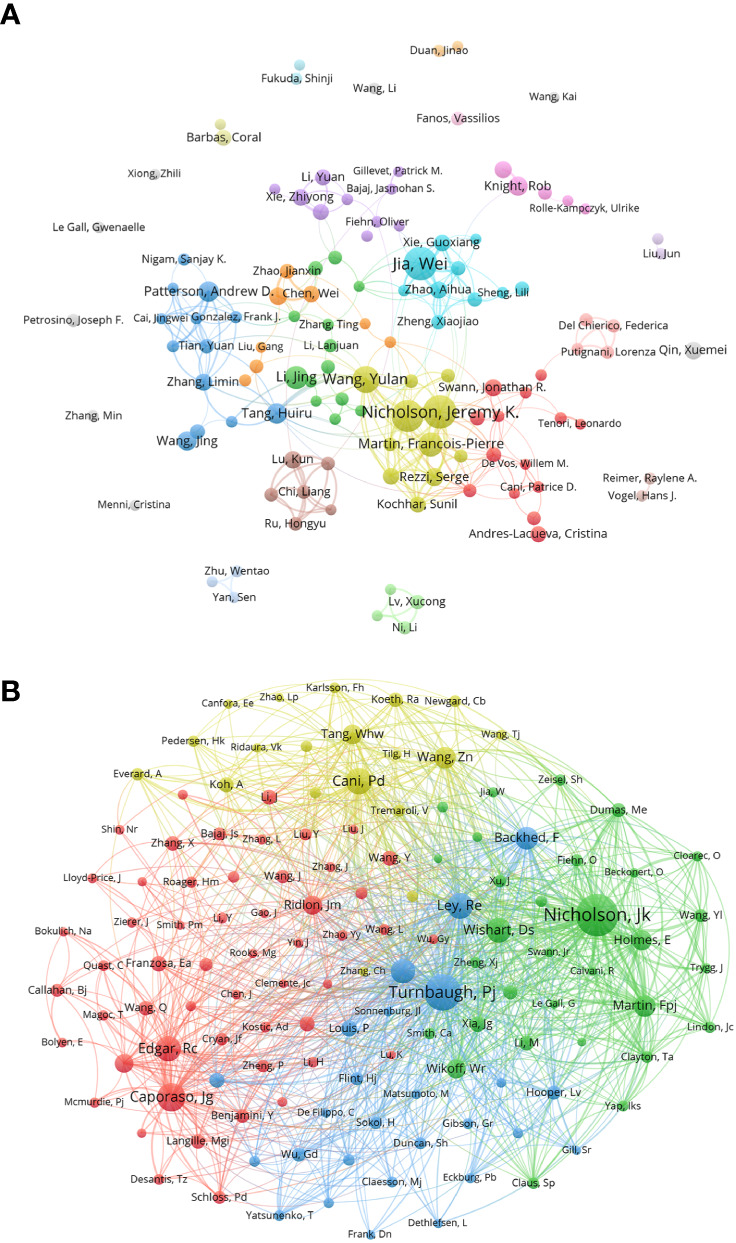
**(A)** Co-author in the field of metabolomics of gastrointestinal microbiome, the author of each circle represent different, connect the circle line reflects the connection between the author, different colors of the network represents the author groups often cooperation. **(B)** Co-cited-author map in the field of metabolomics of gastrointestinal microbiome. The same color indicates that nodes are in the same cluster. Different colored nodes represent the authors of different collaborative relationships. Word size, circle size and connection thickness are positively correlated with co-citation frequency. (From: VOSviewer, CiteSpace doi: 10.3389/fcimb.2023.1196967).

By analyzing the authors, we can identify representative scholars and core research teams in the field. **Table 3** shows the top 10 authors in this field. In terms of the number of publications, the top 10 scholars include Jia Wei, Nicholson, Jeremy K. Holmes, and Elaine had more than 40, and the remaining seven authors have more than 20. Three of the ten are from the United States and three are from China. Jia Wei and Nicholson, Jeremy K. are the scholars with the largest number of published articles in this field, each of them has published 49 articles. Followed by Holmes, Elaine (46) from England. Furthermore, Nichlson, Jeremy K has 35 of H-index, meaning that it were 35 documents published in journals at least 35 of IF under his work, and then Holmes, Elaine has 33 points of H-index. **Figure 2A** shows cluster analysis of co-author. The cooperation of scholars in this field is relatively scattered and has not formed very close and extensive contacts. The yellow network with Nicholson, Jeremy K. as the active point has the widest scope and is in a relatively central position, which proves that his cooperation with other authors is the most extensive and active.

**Table 3:** Top 10 author and co-cited authors related to gut microbiome and metabolism.

Rank	Author	Documents	Countries/regions	institution	Author	co- citations	Countries/regions	institution	Author	H index	Countries/regions	institution
1	Jia, Wei	49	USA	University of Hawaii Cancer Center	Nicholson, Jeremy K.	981	England	Univ London Imperial Coll Sci Technol & Med	NICHOLSON JK	35	England	Univ London Imperial Coll Sci Technol & Med
2	Nicholson, Jeremy K.	49	England	Univ London Imperial Coll Sci Technol & Med	Turnbaugh, Pj	800	USA	University of California System	HOLMES E	33	England	Imperial College London
3	Holmes, Elaine	46	England	Imperial College London	Caporaso, Jg	547	USA	Northern Arizona University	WANG YL	28	Singapore	Nanyang Technological University
4	Wang, Yulan	36	Singapore	Nanyang Technological University	Cani, Pd	487	Belgium	Catholic University of Louvain	JIA W	27	USA	University of Hawaii Cancer Center
5	Martin, Francois-Pierre	31	Switzerland	Nestle Inst Hlth Sci	Ley, Re	480	Germany	Eberhard Karls University of Tubingen	TANG HR	22	China	Fudan Univ Int Ctr Mol Phen
6	Li, Jing	29	China	Chinese Acad Sci	Edgar, Rc	458	Italy	University of Turin	LINDON JC	19	England	Univ London Imperial Coll Sci Technol & Med
7	Patterson, Andrew D.	24	USA	Pennsylvania Commonwealth System of Higher Education (PCSHE)	Wishart, Ds	449	Canada	University of Alberta	MARTIN FPJ	19	Switzerland	Nestle Inst Hlth Sci
8	Tang, Huiru	24	China	Fudan Univ Int Ctr Mol Phen	Qin, Jj	441	China	University of South China	LI J	18	China	Chinese Acad Sci
9	Knight, Rob	23	USA	Univ Calif San Diego	Backhed, F	393	Sweden	University of Gothenburg	REZZI S	18	Switzerland	Nestle Inst Hlth Sci
10	Wang, Jing	23	China	Xi’an Jiaotong-Liverpool University	Wang, Zn	376	USA	University System of Ohio	WANG Y	18	China	Dali Univ

We made a statistical and cluster analysis of co-cited authors in the field of gastrointestinal microbiome and metabolomics, as shown in **Table 3** and **Figure 2B**. The only authors in the top 10 were Nicholson, Jeremy K. Turnbaugh, Pj’s co-citation reached 800 and above, and there were six authors over 400 and two over 300. Meanwhile, of those ten authors, only Nicholson, Jeremy K. Has a total link strength of over 10000. Three of the top 10 co-cited-author are from USA. Specifically, the author with the largest number of co-citations is Nicholson, Jeremy K. (981), followed by Turnbaugh, Pj (800) and Caporaso, Jg (547). In addition, the total link strength of Nicholson and Jeremy K. reached 10,496, ranking first, followed by Turnbaugh, Pj (9,492), and Ley, Re (5675). It is worth noting that Nicholson, Jeremy K. ranks among the top three in both the number of publications and the co-citation, which proves that he has made great contributions and is highly recognized in this field. **Figure 2B** visually illustrates the network of academic partnerships and collaborations in this field. Caporaso, Jg, Cani, Pd, Turnbaugh, Pj and Nicholson, Jeremy K. The most active authors in the red, yellow, blue, and green clusters, respectively, occupy the top four positions in the co-citation rankings. This indicates that the research directions of authors with high co-citation times are almost all different, but the academic relationship between them is still close.

### 3.4 Journals and co-cited-journal

**Figure 3:**
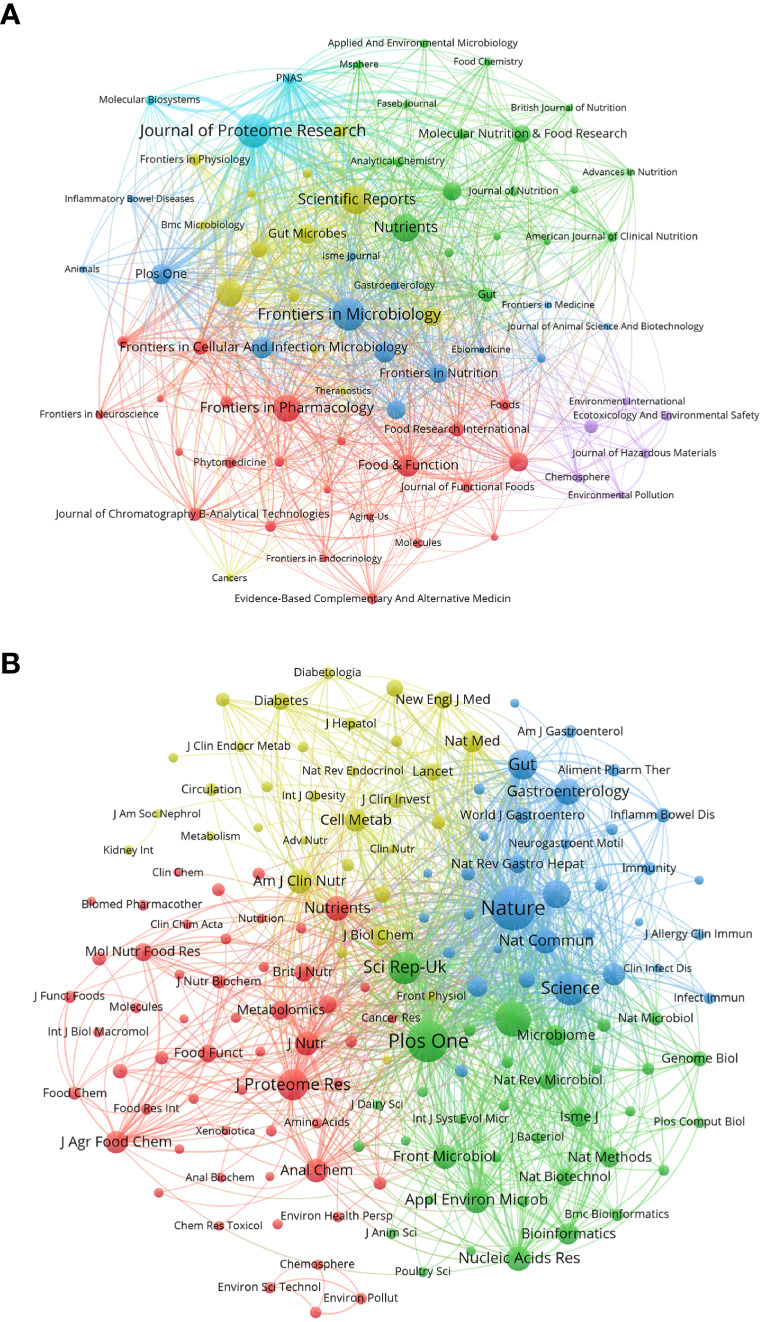
**(A)** Gastrointestinal microbiota and metabonomics related journals. Each dot represents a journal. The same color indicates that the nodes are in the same cluster. Node size and link thickness reflect the frequency of journal cooperation. **(B)** Co-cited-journal analysis related to gastrointestinal microbiology and metabolomics. The same color indicates that the nodes are in the same cluster. The size of nodes reflects the co-citation frequency of journals, and the link represents the co-citation relationship between journals, which is proportional to the thickness of the link. (From: VOSviewer, CiteSpace doi: 10.3389/fcimb.2023.1196967).

We used a bibliometric online analysis platform to identify journals with high publication and impact in the field of gastrointestinal microbiome metabolomics and visualized them programing with VOS viewer. **Table 4** shows the top 10 journals with the number of publications and co-citation, and their corresponding IF (JCR2021) and JCR quartile. The top ten journals are all distributed in Q1 JCR and Q2 JCR, and eight journals have an IF higher than 5. The number of publications of Journal of Proteome Research and Frontiers in Microbiology exceeds 100, indicating that these two journals are more active in this field than other journals. The largest number of publications was Journal of Proteome Research (IF=5.37, Q1) with 131 publications, followed by Frontiers in Microbiology (IF=6.064, Q1) (124 documents) and Scientific Reports (IF=4.997, Q2) (99 copies). In addition, we use cluster analysis to roughly divide all journals into 6 categories as shown in **Figure 3A**. The connections between the entire journal network are very close and extensive. The red cluster includes journals such as Food&Function and Journal of Functional Foods. The yellow area contains Gut Microbiomes and Science Reports, among others. The dark blue cluster contains Frontiers in Microbiology and Frontiers in Cellular and Infection Microbiology. Journal of Proteome Research, which ranks first in the number of publications, and PNAS belong to the sky-blue area. The Green cluster contains journals such as Molecular Nutrition&Food Function and Journal of Nutritiond. The purple area includes journals such as Chemosphere And Ecotoxicology And Environmental Safety.

**Table 4:** The number of publications, IF (JCR2021),and JCR quartile of the top 10 journals and co-cited-journal.

Rank	Journal	Publications	IF(JCR2021)	JCR quatile	Co-Cited-Journal	Citations	IF(JCR2021)	JCR quatile
1	Journal of Proteome Research	131	5.37	Q1	Nature	6944	69.504	Q1
2	Frontiers in Microbiology	124	6.064	Q1	Plos One	6056	3.752	Q2
3	Scientific Reports	99	4.997	Q2	P Natl Acad Sci Usa	4544	12.779	Q1
4	Nutrients	95	6.706	Q1	Science	3883	63.832	Q1
5	Frontiers in Pharmacology	91	5.988	Q1	Sci Rep-Uk	3735	4.997	Q2
6	Metabolites	84	5.581	Q2	J Proteome Res	3376	5.37	Q1
7	Food & Function	64	6.317	Q1	Gut	3173	31.795	Q1
8	Frontiers in Cellular And Infection Microbiology	61	6.703	Q1	Cell	2777	66.85	Q1
9	Frontiers in Immunology	57	8.787	Q1	Gastroenterology	2459	33.883	Q1
10	Plos One	57	3.752	Q2	Appl Environ Microb	2361	5.005	Q2

**Table 4** shows that the top 10 co-cited-journal are all located in Q1 JCR and Q2 JCR. Three out of 10 co-cited-journal had an IF of more than 60. Nature ranked first, with IF=69.504 and citation times up to 6944, followed by Plos One (IF=3.752, with 6056 citations) and PNAS (IF=12.779, with 4544 citations). Plos One is the only journal which ranks in the top 10 in both the number of publications and citations. We can directly observe information such as cooperation between journals through the cluster analysis in **Figure 3B**. J Proteome Res, Am J Clin Nutr, Nature and Plos One were active journals in red, yellow, blue, and green clusters with extensive co-citation relationships, respectively.

### 3.5 Hot spots and frontiers supporting by References and reference burst analysis

**Table 5** shows the top 15 most frequently cited articles, among which only one article cited more than 3,000 times, five cited more than 1,000 times, and the remaining four cited more than 600 times. Additionally, seven of the 10 articles were published in 2015 or earlier. Among them, Wang ZN et al. ‘s paper “Gut flora metabolism of phosphatidylcholine promotes cardiovascular disease” published in Nature in 2011 is the most frequently cited paper. It was cited 3,224 times in total. This article uses metabolomics to demonstrate the key role of gastrointestinal microbiome in TMAO production, macrophage cholesterol accumulation, and foam cell formation. In addition, they demonstrated that gastrointestinal microbiome and gastrointestinal microbiome metabolome are involved in dietary cholinine-induced atherosclerosis (Wang et al., 2011). The second most cited article was “Microbiota Modulate Behavioral and Physiological Abnormalities Associated with Neurodevelopmental Disorders” by Hsiao, EY et al. published in CELL in 2013, with a total of 1880 citations. In this study, the authors “demonstrated barrier defects and microbiome alterations in the maternal immune activation (MIA) mouse model that is known to display features of autistic spectrum disorder(ASD)”. They also demonstrated that oral treatment of MIA offspring with human commensal Bacteroides fragilis could therapeutically restore changes in the gastrointestinal microbiome and its metabolome that occur in MIA offspring, thereby modulating the levels of several serum metabolites and thereby correcting ASD-related behavioral abnormalities (Hsiao et al., 2013). The article published in PNAS “Metabolomics analysis reveals large effects of gut microflora on mammalian blood metabolites” wrote by Wikoff, WR et al. in 2009 ranked second. In this article, the researchers report a broad MS-based metabolomics study that demonstrates a surprisingly large effect of the gut “microbiome” on mammalian blood metabolites. They used various mass spectrometry (MS) based methods to demonstrate a significant interaction between the gut bacterial metabolome and the state and overall metabolism of the organism (Wikoff et al., 2009).

**Table 5:** Top 15 high-cited articles related to gut microbiome and metabolism.

Rank	Author	Article Title	Source Title	Citations	Year	Document Type	DOI
1	Wang, ZN et al.	Gut flora metabolism of phosphatidylcholine promotes cardiovascular disease	NATURE	3224	2011	Article	10.1038/nature09922
2	Hsiao, EY et al.	Microbiota Modulate Behavioral and Physiological Abnormalities Associated with Neurodevelopmental Disorders	CELL	1880	2013	Article	10.1016/j.cell.2013.11.024
3	Wikoff, WR et al.	Metabolomics analysis reveals large effects of gut microflora on mammalian blood metabolites	PNAS	1649	2009	Article	10.1073/pnas.0812874106
4	Markle, JGM et al.	Sex Differences in the Gut Microbiome Drive Hormone-Dependent Regulation of Autoimmunity	SCIENCE	1144	2013	Article	10.1126/science.1233521
5	Johnson, CH et al.	Metabolomics: beyond biomarkers and towards mechanisms	NATURE REVIEWS MOLECULAR CELL BIOLOGY	1073	2016	Review	10.1038/nrm.2016.25
6	Kostic, AD et al.	The Microbiome in Inflammatory Bowel Disease: Current Status and the Future Ahead	GASTROENTEROLOGY	1024	2014	Article	10.1053/j.gastro.2014.02.009
7	Franceschi, C et al.	Inflammaging: a new immune-metabolic viewpoint for age-related diseases	NATURE REVIEWS ENDOCRINOLOGY	925	2018	Review	10.1038/s41574-018-0059-4
8	Plovier, H et al.	A purified membrane protein from Akkermansia muciniphila or the pasteurized bacterium improves metabolism in obese and diabetic mice	NATURE MEDICINE	922	2017	Article	10.1038/nm.4236
9	Dumas, ME et al.	Metabolic profiling reveals a contribution of gut microbiota to fatty liver phenotype in insulin-resistant mice	PNAS	807	2006	Article	10.1073/pnas.0601056103
10	Li, M et al.	Symbiotic gut microbes modulate human metabolic phenotypes	PNAS	802	2008	Article	10.1073/pnas.0712038105
11	Fan, Yet al.	Gut microbiota in human metabolic health and disease	NATURE REVIEWS MICROBIOLOGY	754	2021	Review	10.1038/s41579-020-0433-9
12	Li, J et al.	Gut microbiota dysbiosis contributes to the development of hypertension	MICROBIOME	722	2017	Article	10.1186/s40168-016-0222-x
13	De Filippis, Fet al.	High-level adherence to a Mediterranean diet beneficially impacts the gut microbiota and associated metabolome	GUT	712	2016	Article	10.1136/gutjnl-2015-309957
14	Knight, R et al.	Best practices for analysing microbiomes	NATURE REVIEWS MICROBIOLOGY	674	2018	Review	10.1038/s41579-018-0029-9
15	Nicholson, JK et al.	Gut microorganisms, mammalian metabolism and personalized health care	NATURE REVIEWS MICROBIOLOGY	670	2005	Review	10.1038/nrmicro1152

**Figure 4:**
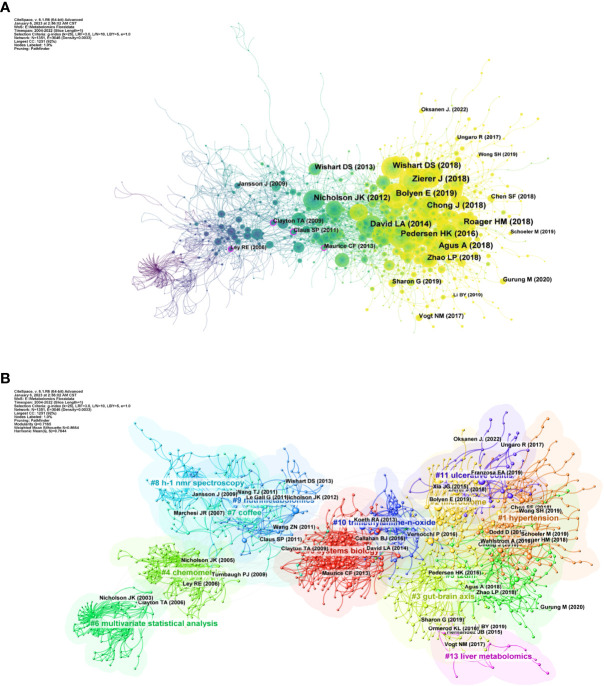
**(A)** References in the field of gastrointestinal flora metabolomics. **(B)** Domain references’ keywords map. The same color indicates that these keywords are in the same clusters, and the links indicate the co-occurrence relationship between the keywords. (From: VOSviewer, CiteSpace doi: 10.3389/fcimb.2023.1196967).

We analyzed the relationship between references using CiteSpace as shown in **Figure 4**. **Figure 4A** shows that before 2010, Jansson et al. (2009), Clayton et al. (2009) and Ley et al. (2006) were the most frequently co-cited. In 2010 onwards, the cluster of references with Nicholson et al., (2012), Wishart et al. (2018), Bolyen et al. (2019), and Zierer et al. (2018) as active points then occupy a significant position in the high frequency co-cited literature. **Figure 4B** shows the clustering relationship mapping of references. The category with the highest number of publications is #0 systems biology, which is more frequently associated with #10 trimethylamine-n-oxide. It is followed by #1 hypertension, #2 microbiome and #3 gut-brain axis. In terms of temporal dimension, the earliest studied topic in the field of metabolomics of the gastrointestinal microbiome was #6 multivariate statistical analysis, of which the main citations were published by Nicholson JK et al. in 2003 and Clayton TA et al. in 2006. In addition, #4 chemomet, #8 h-1 nmr spectroscopy, and #9 nutrimetabolomics are all early clusters of interest in this area. They all have cross-cited articles with each other. The emerging high co-citation topics are #1 hypertension, #5 t2dm, #11 ulcerative colitis and #3 gut-brain axis, which are closely related to each other and the authors of high-cited articles in these fields have collaborated more. This also indicates that research related to these topics is a recent hot topic in the field and at the forefront of research. The current high topics are #1 hypertension, #t2dm, #11 ulcerative colitis, #3 gut-brain axis, and #13 liver metabolomics which are closely related to each other and the authors of the top references in these fields have collaborated more. This also indicates that research related to these topics is a recent hot topic in the field and at the forefront of research.

**Figure 5:**
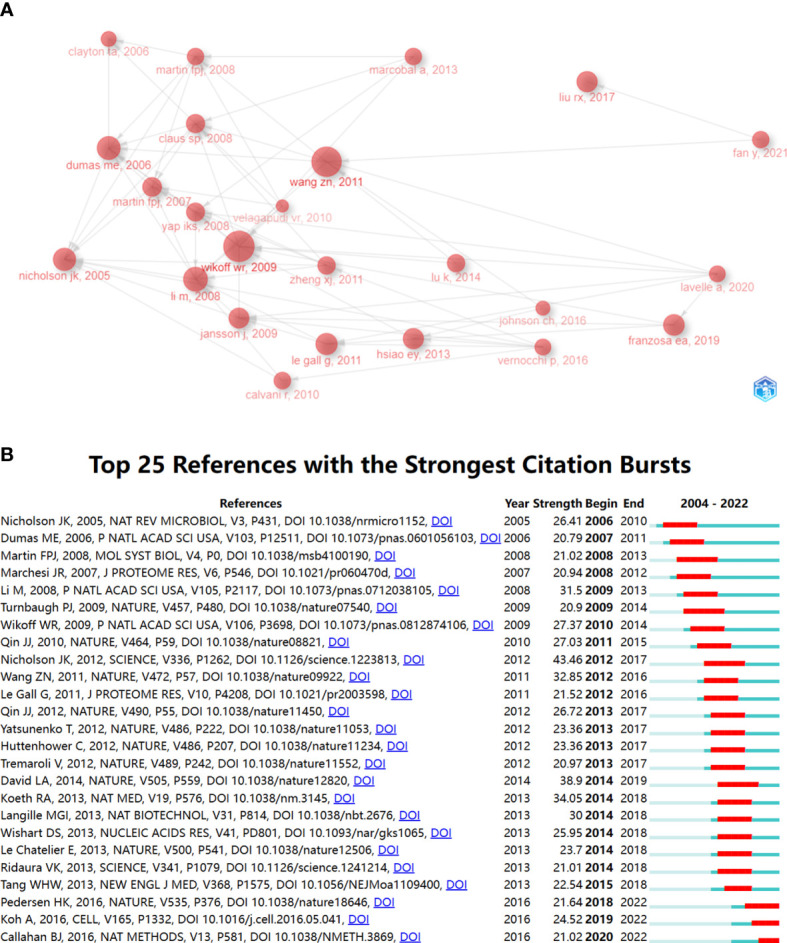
**(A)** Relationship graph of high cited articles, the connecting lines represent the citations between them. sizes of nodes mean citations of documents. **(B)** The top 25 references involving the strongest citation burst in gastrointestinal microbiota metabolomics, sorted by year of origin, the blue bars mean the reference had been published, the red bars represent citation burstness.

Briefly, a more concise analysis of high cited references shows clearly the structure of development (**Figure 5A**). The earliest high cited article is “Gut microorganisms, mammalian metabolism and personalized health care” Nicholson, JK published in 2005, arguing necessity of gut microbiome with it metabolites in future human health care (Nicholson et al., 2005). The most cited article and core node is “Metabolomics analysis reveals large effects of gut microflora on mammalian blood metabolites” Wikoff, WR published in 2009, revealing the important role of gut microbiome in on blood metabolites (Wikoff et al., 2009). In last years, “Gut microbiota in human metabolic health and disease” Fan, Y published in 2021 reviews the mechanism of gut microbiome and metabolites in human metabolic diseases. Two resemble work lavelle, A et al. and franzosa, EA et al. finished respectively to inflammation bowel diseases are published as “Gut microbiota-derived metabolites as key actors in inflammatory bowel disease” in 2020 (Lavelle and Sokol, 2020) and “Gut microbiome structure and metabolic activity in inflammatory bowel disease” in 2019 (Franzosa et al., 2019).

**Figure 5B** shows the top 25 references with the strongest citation burst. Between 2005 and 2015, there were one or more citation burst every year, which lasted for about five years. The next time of citation burst after 2015 took place in 2018, and there are still new articles in the next year. It is noteworthy that there are six times of citation burst occurred in 2014 and all continued into 2018 and even 2019, the highest number of burst times in recent years. The first time that citation burst in the field occurred in 2006. The article “Gut microorganisms, mammalian metabolism and personalized health care” was published in 2005 by Nicholson JK, a key figure in the field, in NAT REV MICROBIOL. and this burst continued into 2010. The present topic of references with strong citations focuses on common endocrine system diseases represented by type 2 diabetes and hypertension, while some scholars are still studying the effects of diet and gut microbiome metabolome on the host(Koh et al., 2016; Pedersen et al., 2016; De Filippis et al., 2019; Das et al., 2021).

### 3.6 Hot spots and frontiers supporting by Keywords analysis

**Figure 6:**
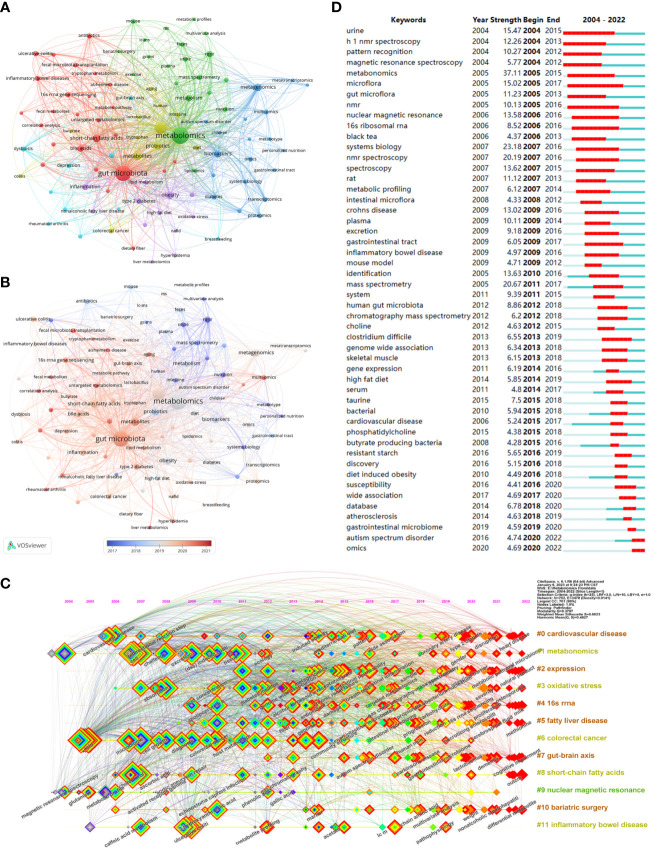
**(A)** Cluster diagram of keyword co-occurrence analysis network. Keywords are divided into 6 clusters. Each keyword is represented as a node, and the node size is proportional to the frequency. The lines between nodes indicate co-occurrence relationships. The distance and intensity between nodes indicate the relevance. **(B)** Co-occurrence network analysis graph of keywords, different colors represent different mean published years. **(C)** Timeline view of co-cited literature related to metabolomics of the gastrointestinal microbiome. Each horizontal line represents a cluster, node size reflects co-citation frequency, links indicate co-citation relationships, and colors of nodes and lines indicate different years. **(D)** The fifty keywords with the strongest citation burst in gastrointestinal microbiome metabolomics, sorted by starting year, the blue bars mean the reference had been published, the red bars represent citation burstiness. (From: VOSviewer, CiteSpace doi: 10.3389/fcimb.2023.1196967).

Keywords are an overview of the core content of an article, so we can use it to analyze the research hotspots and frontiers in the field of metabolomics of gastrointestinal microbiome. The top 20 keywords in the field are shown in **Table 6**. Two keywords had more than 1000 occurrences and nine had more than 100. Three of them had more than 1000 total link strength and the rest had more than 100. As keywords included in the search formula, metabolomics (1554), gut microbiota (1249), microbiome (632), and microbiome (172) occupied the top 4 positions for the keyword occurrences. We then use VOSviewer to perform a cluster analysis of keywords as a basis for summarizing research themes and hotspots to help scholars understand the direction and trends of research priorities in the field, and to clarify the relationships between different topics in the field.As can be seen in **Figure 6A**, The entire cluster analysis network is highly connected with strong co-occurrence relationships, and there are many node keywords such as metagenomics and short-chain fatty acids that play an important role in bridging other branches of the field. The red cluster contains small molecules associated with gastrointestinal microbiome and with substances associated with the gut (bile acids, short-chain fatty acids, tryptophan, etc.). The yellow clusters are associated with some other diseases related to the variation of the gastrointestinal microbiome (colitis, autism spectrum disorders, etc.). The keywords in the blue cluster are related to various histologies and their biomarkers (biomarkers, macrogenome, proteomics, transcriptome, etc.). The light blue cluster concentrates keywords related to ecological disorders and inflammatory diseases of the human body (ecological disorders, depression, rheumatoid arthritis, inflammatory diseases, etc.). The green cluster clusters keywords related to metabolism and metabolomics research techniques (metabolism, feces, urine, ms, gc-ms, lc-ms, etc.). The purple cluster clusters keywords related to metabolism-related nutrition and diseases (type 2 diabetes, obesity, diet, dietary fiber, etc.). **Figure 6B**, on the other hand, shows the hot keywords of different years with different colors based on the keyword clustering analysis. The most hot keywords in average published year are serum metabolomics, correlation analysis, and fecal metabolites in red cluster; exercise, bariatric surgery, and MS in green cluster; multi-omics, machine learning, and proteomics in blue cluster; aging, metabolites, and colitis yellow cluster; liver metabolomics, hyperlipidemia, and lipid metabolism purple cluster; and rheumatoid arthritis, 16s rrna, and fecal metabolomics in light blue cluster.

**Table 6:** Top 20 keywords with the highest occurrence times and their total link strength.

Rank	Keyword	Occurrences	Total link strength	Rank	Keyword	Occurrences	Total link strength
1	metabolomics	1554	2917	11	bile acids	101	236
2	gut microbiota	1249	2124	12	metabolism	99	197
3	microbiome	632	1246	13	nmr	91	214
4	metabolites	172	343	14	type 2 diabetes	65	147
5	metagenomics	161	455	15	16s rrna gene sequencing	63	153
6	short-chain fatty acids	157	354	16	inflammatory bowel diseases	63	161
7	obesity	150	397	17	mass spectrometry	59	147
8	biomarkers	133	281	18	ulcerative colitis	58	132
9	probiotics	128	286	19	diet	55	150
10	inflammation	106	246	20	transcriptomics	55	168

**Figure 6C** visualizes the stage research hotspots and development direction of metabolomics of gastrointestinal microbiome from the time dimension. Combining these two charts we can understand the evolutionary trajectory and stage characteristics of this field. As shown in the figure, the keyword clusters that started the research boom early were #1 metabonomics, #0 cardiovascular disease, and #6 colorectal cancer. The cluster #9 nuclear magnetic resonance, despite a small amount of research in 2004-2006, and being one of the first keyword clusters in the field, has been low and declining. and was one of the first keyword clusters to appear in this field, it has been less hot and declining. It is worth noting that 10 of the 12 clusters continue to be studied (#0 cardiovascular disease, #1 metabonomics, #2 expression, #3 oxidative stress, #4 16sRNA, #5 fatty liver disease, #7 gut-brain axis, #8 gut-brain axis, and #9 gut-brain axis, #7 gut-brain axis, #8 short-chain fatty acids, #10 bariatric surgery, and #11 inflammatory bowel disease).

**Figure 7:**
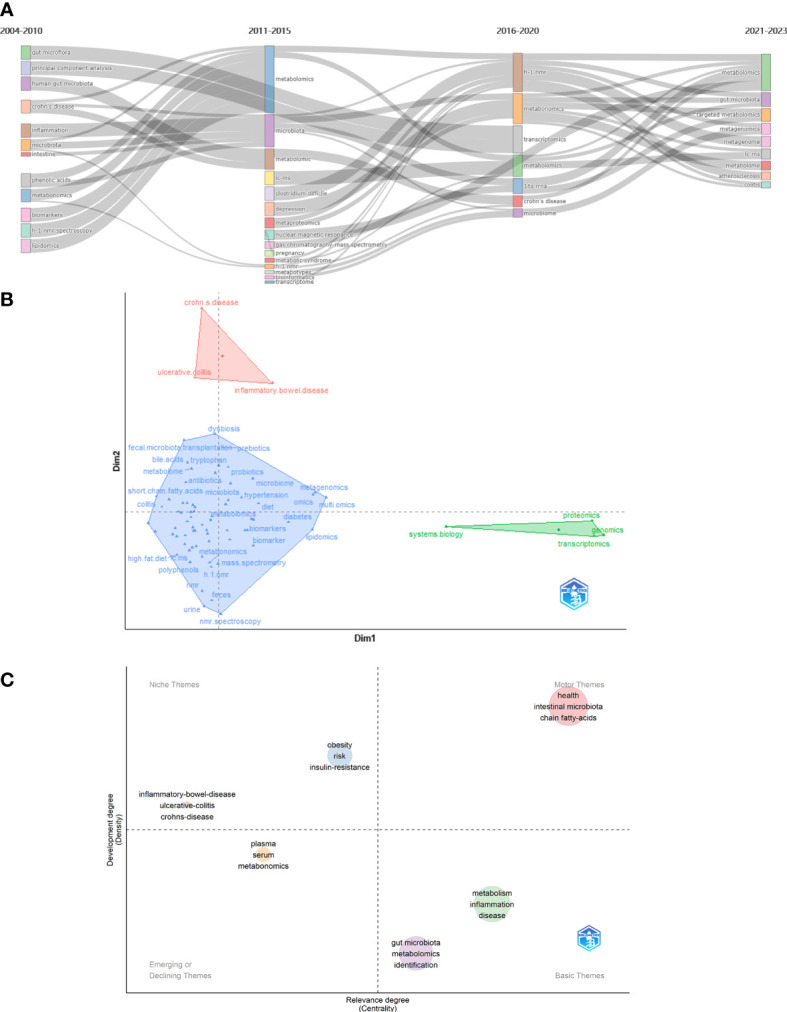
**(A)** Thematic evolution cutting in 4 phases. Keywords are ranged according lengths of keywords representing occurences. **(B)** Conceptual structure map using method MCA. Different colors mean different clusters. **(C)** Thematic map. Different colors mean different clusters, and X axis shows relevance degree as well as Y axis shows development degree.

Keyword with strong citation burst can also reflect some emerging academic trends and hot topics in the field, as well as being used to predict cutting-edge research directions and reveal potential hot spots in the field. **Figure 6D** lists the top 50 keywords with the strongest citation bursts. The earliest was urine, followed by h 1 nmr spectroscopy and pattern recognition, while the keywords in recent years are autism spectrum disorder and omics, which represent the current hot spots and frontiers in the field of gastrointestinal microbiome and metabolomics. According Bibliometrics, to all the keywords, as the most influential keywords, it were principal component analysis, crohn’s disease, and inflammation from 2004 to 2010;lc-ms, clostridium difficile, and depression from 2011 to 2015; h-1 nmr, transcriptomics, and 16s rrna from 2016 to 2020; and targeted metabolomics, metagenomics, and metagenomice from 2021 to 2023, without gut microbiome, metabolomics, or other counterparts (**Figure 7A**).

Method Multiple Correspondence Analysis(MCA) finds two dimensions to describe the distribution of keywords (**Figure 7B**). In diverse three clusters, green cluster has keywords of system biology, proteomics, genomics, and transcriptomics, merely showing characterize of dim1; and red cluster has crohn’s disease, ulcerative colitis, and inflammtory bowel disease, merely showing only the characterize of dim2. Contrarily, blue cluster seem to be indifferent. In **Figure 7B**, the secondary analysis display a theme map that cluster of inflammatory bowel disease, ulcerative colitis, and crohn’s disease situates in realm of niche themes, as well as cluster of obesity, risk, and insulin resistance, while health, intestinal microbiota, and short chain fatty-acids situate in motor themes; metabolism, inflammation, and disease situate in basic theme, as well as gut microbita, metabolomics, and identifications; and plasma, serum, and metabonomics situates in emerging or declining themes.

**Figure 8:**
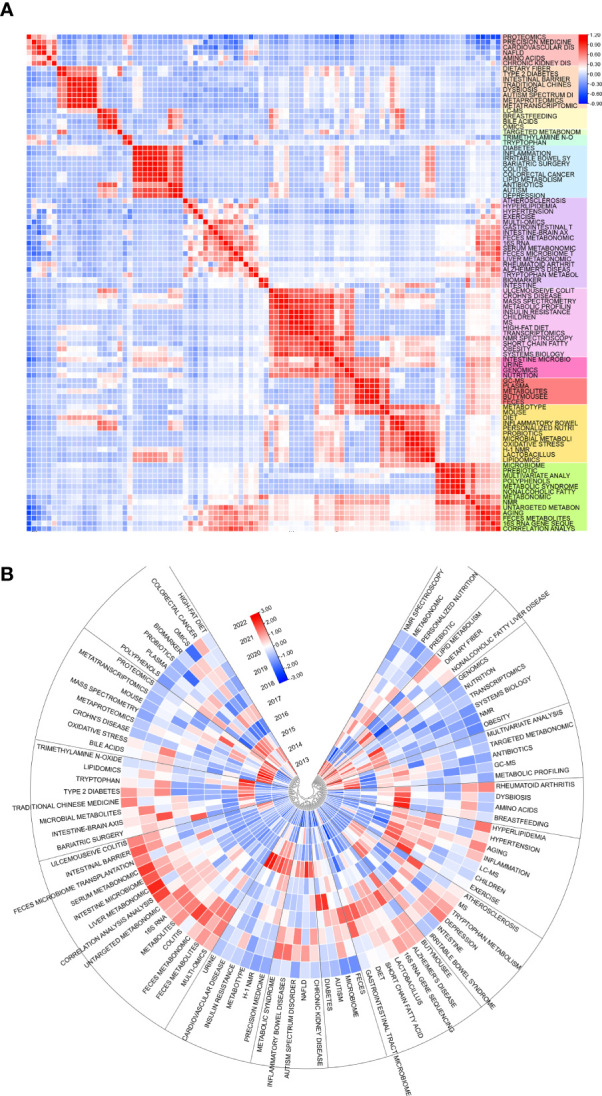
**(A)** Heat map of keywords themselves and **(B)** time-related heat map of keywords. Sections discrete with colors or blocks mean clusters of keywords. Top 100 keywords are counted.

Limited to top 100 keywords and using heart map analysis to co-occurences, some visibly clusters floated in our eyesight(**Figure 8A**). Amino acids have seem studied closely with NAFLD, CKD, cardiovascular disease, and proteomics. Breastfeeding takes relation with bile acids, omics, and LC-MS, while a connection building in range from diabetes to depression. At the meantime, 4 clusters could identified from ulcemouseive colitis to correlation analysis but branches were shared to each adjacent clusters. Besides, it seem not to be remarkable cluster from atherosclerosis to intestine. The correlation depending times both shows some clusters displayed the resemble trend of inner keywords(**Figure 8B**). There are nmr spectroscopy, metabonomic, personalized nutrition and prebiotic turn to be cold in early hot keywords, but not lipid metabolism, dietary fiber, and NFLD, which are revived in some degree. The hot keywords in current are included in cluster of ulcemouseive colitis, intestinal barrier, and liver metabonomic; type 2 diabetes, traditional chinese medicine, and gut- brain axis; alzheimer’s disease, 16s rna gene sequencing, and lactobacillus; and hyperlipidemia, hypertension, and aging.

## 4 Discussion

### 4.1 General information

In this study, we performed a bibliometric analysis of articles in the field of gastrointestinal microbiome metabolomics from 2004 to 2022 and used VOS viewer and CiteSpace to visually analyze and present the evolutionary trends and research frontiers hotspots in the field.

From the results of our analysis, the number of citations and publications in the field of gastrointestinal microbiome metabolome has continued to increase in recent years. China, USA, and England are the top contributors in this field. China is the country with the highest number of publications. USA is the country with the highest citation frequency and the most extensive collaboration with other countries or regions. **Figure 2** shows that five of the top ten institutions in terms of number of publications are from China, with Chinese Acad Sci being the most productive institution in the institutional collaboration network and providing the highest total link strength. the highest number of citations is from Univ London Imperial Coll Sci Technol & Med. This is probably due to the academic support provided by USA to their national researchers, which has helped them to make good progress in the field(Turnbaugh et al., 2007; The Integrative, 2019), Chinese institutions have also provided significant political and fund support to scholars conducting in-depth research in this area.

Journal of Proteome Research is the top journal in terms of number of publications while Nature is the top journal in terms of co-citations. We can find that the top 10 journals in terms of number of publications and co-citations are focused on different research priorities, which indicates that metabolomics of gastrointestinal microbiome can be widely cited in various fields of basic exploration. Wang ZN et al. published in Nature in 2011”Gut flora metabolism of phosphatidylcholine promotes cardiovascular disease” (Wang et al., 2011) is the highest co-cited article, which indicated that scholars in the field are paying more attention to this article. Nicholson, Jeremy K. is the number one co-cited-author and he is tied with Jia, Wei for the first number of publications in this field with 35 of H-index, and he coauthored the first co-citation outbreak in this field, showing his outstanding influence and significant contribution in the field of gastrointestinal microbiome and metabolomics. In recent years, Wishart DS et al. not only have gradually become central figures in the field but also have a high citation. Their research focus can also reflect the current research trends in the field. For example, Wishart DS et al. updated the human metabolomics database in 2022 (Wishart et al., 2022), which provides an effective means for further exploration of the field. In conclusion, the field continues to evolve rapidly as new researchers emerge and the important discoveries they bring.

**Figure 9:**
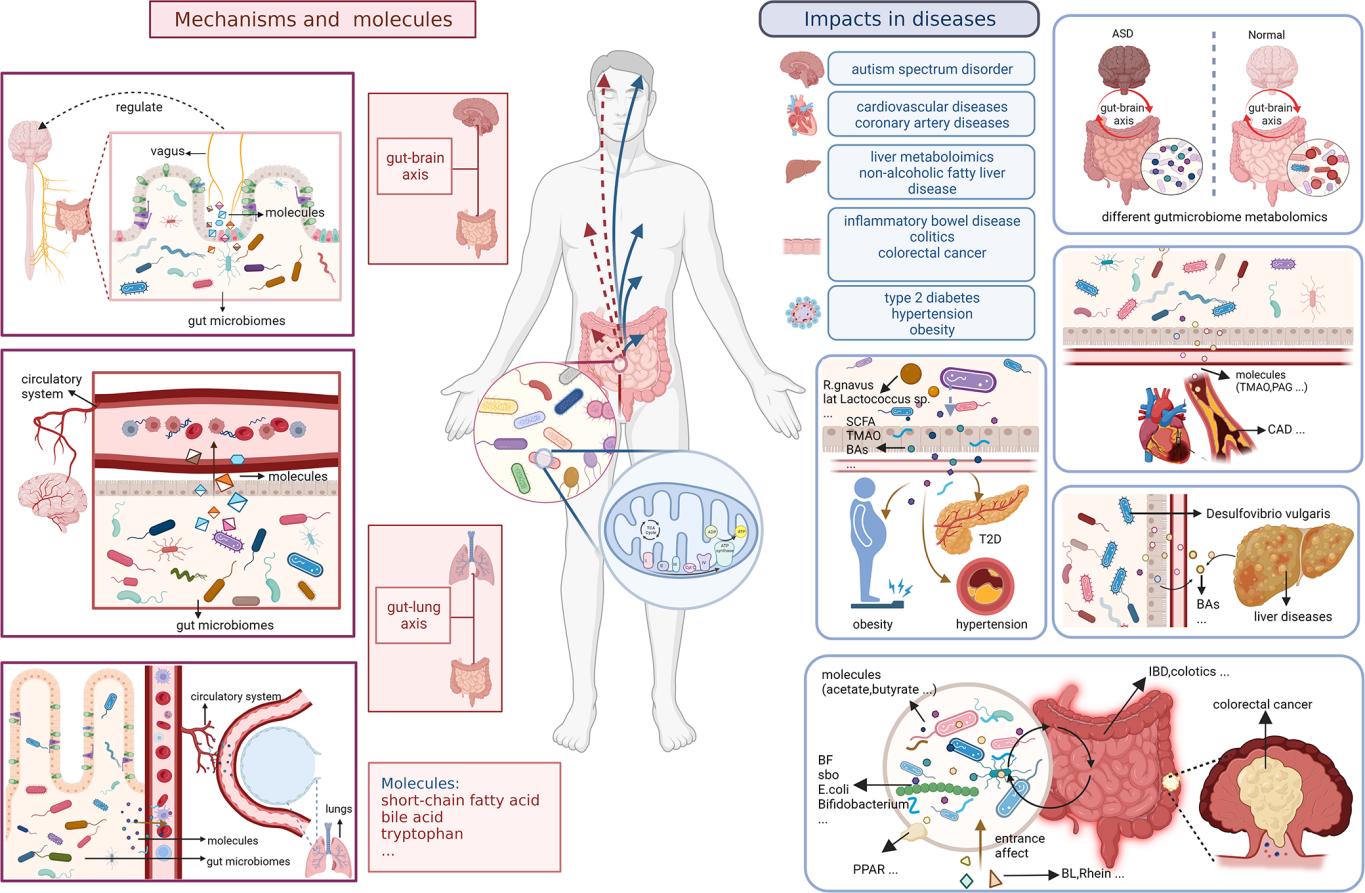
Small molecules and specific mechanisms related to gut microbiota and metabolism in body and diseases.

We have also analyzed the overall evolutionary trends in the field. From a keyword perspective, metabolomics, and gut microbiota, as members of the search formula, have been the core, focus and foundation of the research content of the entire field. It is worth noting that keywords related to small molecules such as metagenomics and acids play an important role as a bridge to other branches of the field. This suggests that there is a strong research potential for them to co-investigate the field with other interdisciplinary disciplines, and that scholars can also start to explore more in these areas. The frequently occurring keywords in different time periods can also reflect the emerging academic hotspots in the field and show the evolutionary trends in the field. In terms of key topics of articles, #1 metabonomics and #0 cardiovascular disease are the larger clusters of keywords that appear earlier. Fatty liver diseases, colorectal cancer, and T2D are emerging keywords and keyword clusters. From this point of view, scholars have started to study the link between the metabolomics of the gastrointestinal microbiota and the state of the organism in the direction of diseases and their interventions in 2004, and the enthusiasm of these keyword clusters has continued at a high level until now (**Figure 9**).

### 4.2 Hotspots and frontiers

The analysis of hot keywords and topics helps researchers to keep abreast of current research trends and frontiers in the field, which is crucial in such an era of information explosion. Outcomes in **Figure 7** shows that though the most keywords seem indifferent to characterize with metabolomics methods like transcriptomics directly, the prevail technology of metabolomics have iteration in several times to adapt the development of needing to advance, from LC-MS to H-1 NMR, from transcriptomics to metagenomics. We summarize the hot topics in the academic field and objectively evaluate the frontier research directions in the field of gastrointestinal microbiome metabolomics through the analysis of bibliometric and visualization software, then divide them into two categories: the exploration of specific mechanisms and related metabolic small molecules, and the impact of gastrointestinal microbiome metabolomics in various diseases.

#### 4.2.1 Specific mechanisms and molecules

Small molecule metabolites of the gastrointestinal microbiome such as short chain fatty acids (SCFA), bile acids and tryptophan metabolites play an important role in the bidirectional communication of the gastrointestinal microbiome metabolome with the host through direct brain exposure, enterochromatic cell stimulation and immune communication (Franzosa et al., 2018; Nicolas and Chang, 2019; Krautkramer et al., 2020). SCFA can influence host organism status through immune communication such as regulating the intestinal T-cell pool and influencing intestinal B-cell responses (Lavelle and Sokol, 2020). Marchesi JR et al. explored changes in the levels of phenotypic metabolites such as SCFAs in patients with inflammatory bowel diseases including Crohn’s disease and ulcerative colitis by using a non-invasive metabolomics approach. Simultaneously, they described the correlation between changes in intestinal flora and changes in metabolomic small molecules (Marchesi et al., 2007). Many intestinal bacterial species modify the primary bile acids of host cholesterol metabolism to secondary bile acids through metabolic mechanisms such as enzymes, based on which the circulation and signaling of bile acids are altered, etc. This effect affects many body functions through the gut-brain axis and contributes to the development of diseases such as Parkinson’s disease, multiple sclerosis and Alzheimer’s disease (Hertel et al., 2019; Nho et al., 2019; Bhargava et al., 2020). Hu H et al. demonstrated that Vibrio desulfuricans in the intestinal microbiome promotes gallstone formation through its effects on bile acid and cholesterol metabolism. The increase in Clostridium perfringens contributes to the transitional excretion of fecal bile acids, providing a mechanistic hypothesis for the study of diarrheal irritable bowel syndrome that can be tested for clinical significance (Zhao et al., 2020).

In recent years there have been many studies dedicated to explore the specific link between the metabolomics of the gastrointestinal microbiome and the gut-brain axis. The gut microbiome metabolome can influence the development and function of the body’s immune, metabolic and nervous systems through gut-brain signaling in many different ways (Cryan et al., 2020; Needham et al., 2020). Some scholars have also studied the specific impact mechanisms. For example, Sgritta M et al. found that Lactobacillus and bacterial metabolites regulate oxytocin levels in the host brain in a vagal-dependent manner (Sgritta et al., 2019), thus participate in many *in vivo* biochemical processes through methods such as metabolomics. In addition, Lactobacillus and its metabolites are involved in regulating many other physiological and psychological processes by modulating the expression of GABA receptors in the brain *via* the vagus nerve (Bravo et al., 2011). Butyrate, a SCFAs, was found a function to synthesizing acetone bodies in liver may to provided energy to brain. DKA children could be detected the accumulation of beta-hydroxy butyrate (Wootton-Gorges et al., 2005). What’s more, according metabonomics method, it shows diverse role in regulating activities of brain. it is unclear or little that butyrate acts on brain directly. However, butyrate could stimulate enteroendocrine cells(EEC) to release neuropeptide and neurotransmitter like serotonin in role of controlling immune cells like Treg cell in anti-inflammation, else, and regulating nervi vagus to adjust permeability of intestinal barrier and blood-brain barrier(BBB) for better regulation of interaction among gut microbiome, metabolism, and human body(Stilling et al., 2016). Furthermore, butyrate is a key to reduce inflammation, improve macrophage development and restrain the excessive growth of intestinal epithelium by inhibting histone deacetylase(HDAC) (Salvi and Cowles, 2021; Liu et al., 2023; Park et al., 2023). Butyrate is supported to influence the growth of glioblastoma acrossing BBB (Lyu et al., 2022). When unbalanced diet coming, the changes of gut microbiome are terrible. There would be production of bile acids, NOCs, and H_2_S to cause DNA damage, amplify oxidative stress, exacerbate gut break, and then H_2_S could reduce SCFAs including butyrate. Above all, in abnormal way, injured microbiome may produce carcinogen, reduce SCFAs, lead to unbalance of gut-brain axis to preserve internal environment with effect intestinal barrier, and permit damaged cells in uncontrolled, even deteriorated to cancer like colorectal cancer probably (Song and Bai, 2021).

It has also been demonstrated that the gut microbiome metabolome plays a key role in the gut-lung axis, which is also important for studying the pathogenesis of diseases such as acute lung injury. Hashimoto, Yaeko et al. found that antibiotic cocktail (ABX) induced gastrointestinal microbiome depletion would affect its metabolism and prevent LPS-induced acute lung injury *via* the entero-lung axis (Hashimoto et al., 2022). The gastrointestinal microbiome metabolome also has a potential role in COPD (Bowerman et al., 2020). Nevertheless, such studies have not yet been able to detect specific microbiome metabolomics, and further precise studies are needed in the future to provide new insights into more relevant mechanisms of the lung-gut axis.

In conclusion, these studies provide some theoretical support for the development of future microbial tools targeted for various applications in the gastrointestinal microbiome metabolomics (Raes, 2023). These works also demonstrate that linking gastrointestinal microbiome to small molecule metabolites could provide new tools for the diagnosis or differential diagnosis of many diseases and improve our understanding of their mechanisms. Yet most of the experiments in this kind of literature are designed horizontally, and it is difficult to explain the specific causal mechanism vertically. Therefore, researchers need to explore more deeply and design more longitudinal and comparative experiments.

#### 4.2.2 Impacts of gut microbiomes metabolitics in various diseases

At present, various diseases and microbiomes that have different relationships with metabolism are being studied extensively. Many scholars have used metabolomics tools to deeply explore the gastrointestinal microbiome of people suffering from these diseases, and the research direction in this field is gradually evolving towards it. Moreover, these targeted studies can also provide more potential directions and approaches for the treatment and prognosis of these diseases.

Cardiovascular disease has been widely studied as a hotspot in this field. Yoshida N. et al. discovered the relationship between specific gastrointestinal bacteria: Bacteroides vulgatus and Bacteroides dorei and their metabolome with CAD (Yoshida et al., 2018). Some scholars have further explored the specific related metabolites. TMAO, a metabolite formed by the metabolism of dietary phosphatidylcholine by gastrointestinal microbiome, was proposed in the study of Chelsea L. Organ et al., which may have the effect of producing inhibitors on cardiac function and structure (Organ et al., 2020). In order to determine the specific influence of gastrointestinal microbiome metabolomics on CAD production and whether it can be used as a diagnostic marker, Ottosson Filip et al. used liquid chromatography-mass spectrometry and 16s RNA sequencing to analyze gastrointestinal microbiome and their metabolites to investigate their association with CAD risk (Ottosson et al., 2020). They found that phenylglutamine (PAG) was associated with an increased risk of future CAD and was relatively independent. Talmor-Barkan Y et al. conducted a comprehensive clinical and multiomics analysis of patients with acute coronary syndrome, including serum metabolomics and gastrointestinal microbiological data, to reveal the metabolic deviations and differences in microbiological characteristics between the affected individuals and the control group (Talmor-Barkan et al., 2022). These reports highlight the relationship between gastrointestinal microbiome metabolome and CAD, as well as provide a theoretical basis and new direction for future studies to understand the interactions of host gastrointestinal microbiome metabolome in atherosclerotic disease mechanisms. However, the specific mechanisms of interaction between coronary heart disease and gastrointestinal microbiome, as well as the role of gastrointestinal microbiome metabolome, have not been fully studied. Thus, the specific pathways and molecular mechanisms involved are of great significance for further exploration.

The gastrointestinal microbiome and its metabolome have been found to be abnormal in type 2 diabetic patients and have been explored in depth by many scholars in this field. Zhao LP et al. investigated the association between abnormal gut microbiome and type 2 diabetes mellitus through a randomized clinical study combined with an histological approach (Zhao et al., 2018). Liu Y et al. found that exercise-induced alterations in the metabolome of the gut microbiome, such as short-chain fatty acid biosynthesis and branched-chain amino acid catabolic capacity, were strongly associated with improvements in glucose homeostasis and insulin sensitivity profiles (Liu et al., 2020). Yifei Zhang et al. researched and reported the effect of berberine on T2D by inhibiting bromelukococcal-mediated biotransformation of bile acid deoxycholic acid by means of metabolomics (Zhang et al., 2020). Nevertheless, studies addressing type 2 diabetes and the gastrointestinal microbiome metabolome are currently less used in clinical practice. Moreover, type 2 diabetes has individual variability, and geography, diet and exercise status are important variables common to both T2D and gastrointestinal microbiome metabolome, which may lead to less accurate study results (Whang et al., 2019; Herrema and Niess, 2020; Martínez-López et al., 2022). Thus, there is a need for researchers to develop more sensitive research tools to precisely investigate the mechanisms of interaction between type 2 diabetes and the metabolome of the gastrointestinal microbiome, and to apply them widely in clinical practice.

The metabolic changes in the organism of hypertensive patients are also closely related to the dysregulation of the gastrointestinal microbiome and metabolome. Yan XF et al. demonstrated that high salt- induced hypertension had a significant effect on the metabolome of gastrointestinal microbiome, including the microbiome metabolites corticosterone and indole derivatives, etc.(Yan et al., 2020). Calderon-Perez, Lorena et al. analyzed the levels of microbial metabolites such as SCFA and TMAO in hypertensive patients through comprehensive gut microbiome analysis and targeted metabolomics in their study. A classification method based on specific gut microbes such as SCFA producer Faecalibacterium prausnitzii, Roseburia hominis, and their metabolites was also proposed in their paper (Calderón-Pérez et al., 2020). Therapeutic approaches based on intestinal probiotics such as bifidobacterium and Lactococcus and their metabolites SCFAs and TMAO have gradually begun to be widely studied. Researchers try to reduce the blood pressure of hypertension patients through these approaches to achieve the purpose of improving the body condition. Nevertheless, clinical correlation analyses in hypertensive patients are often influenced by variables that are not strictly uniform, such as age and environment(Muralitharan et al., 2020). In addition, the detailed role of gastrointestinal microbiome metabolomics in the pathogenesis of other types of hypertensions is still not fully understood, so this field has a promising research prospect in hypertensive diseases.

Obesity is also one of the most important diseases associated with the metabolomics of the gastrointestinal microbiome that pose a health burden to the population. Gastrointestinal microbiome can regulate lipid metabolism through the bile acid pathway and thus influence the development of obesity (Mullish et al., 2018). The intake of various types of proteins and short-chain and branched-chain fatty acids in the diet also has a significant impact on the gastrointestinal microbiome and the metabolic group of the intestinal microbiome, which affects the lipid metabolism and blood lipid levels of the body, and is also important for the development of obesity (Prokopidis et al., 2020). Specific bacteria in the gut and their metabolome also impact the progression of nonalcoholic fatty liver disease (NAFLD). Hong,F et al. demonstrated that Desulfovibrio vulgaris is a potent acetic acid-producing bacterium that attenuates NAFLD in mice (Hong et al., 2021). Interestingly, Adams LA et al. also reported in their article that the gut microbiome is a high-risk causative factor for NAFLD (Adams et al., 2017). In this group of people, key physiological functions of the liver, such as lipid metabolism, are disturbed, and this physiological alteration has anindependently association with type 2 diabetes. Choucair I et al. even developed a new novel method to quantify the involvement of gut microbiome in the production of structure-specific bile acids (BAs) in humans and mice, to study their effects on multiple metabolic pathways in the host, and to perform a clinical case-control study of biochemical indicators such as BAs synthesized by the liver in type 2 diabetic patients compared to non-diabetic patients (Choucair et al., 2020). In summary, academic topics related to liver metabolism are also among the potential topics with in-depth exploration.

Gut microbiome metabolomics also plays an essential role in the pathogenesis of inflammatory bowel disease. By studying the association between gastrointestinal microbiome and host metabolism, Bian X et al. identified a potential probiotic agent capable of ameliorating colitis, an inflammatory bowel disease (IBDs) formed by the interaction of genes related to gastrointestinal microbiome and other influencing factors(Bian et al., 2019). There are also other small molecules that can influence the progression of IBD by modulating the metabolome of the gastrointestinal microbiome. For example, Jiawei Wu et al. demonstrated in their report that Rhein, one of the major components of rhubarb, is able to regulate the intestinal microbiome, and the intestinal microbiome is able to alleviate colitis by regulating its own purine metabolism (Wu et al., 2020). Similarly, barley leaf (BL) can also improve intestinal mucosal barrier function by activating peroxisome proliferator-activated receptor (PPAR) γ signaling and thereby increasing intestinal flora purine metabolism levels, and the gut microbiota-inosine-A(2A)R/PPAR γ axis plays an important role in this regulatory mechanism (Li et al., 2021). Yizhong Wang et al. then explored alterations in the metabolome of intestinal flora in pediatric Crohn’s disease patients, such as reductions in SCFAs concentrations and an imbalance of unconjugated/conjugated BA ratio, which can lead to digestive inflammation and mucosal damage (Wang et al., 2021). Although gastrointestinal microbiome and metabolomics have been shown to play an important role in regulating host energy metabolism, immune homeostasis and mucosal integrity in inflammatory diseases of the digestive system represented by IBD(Lee-Sarwar et al., 2020), the exact nature of the changes in commensal flora in inflammatory diseases of the digestive system and the mechanisms of regulation remain to be clarified.

Fukuda S et al. proved in 2011 that the occurrence and progression of intestinal infectious diseases could be inhibited by acetate produced by bifidobacterium in gastrointestinal microbiome through modeling and integrative omics (Fukuda et al., 2011). Marchesi JR et al. reported in their publication on the dynamic interaction between gut microbial ecology and CRC, which provides useful clues for the diagnosis, intervention and treatment of CRC(Marchesi et al., 2011). In their review, Fung KY et al. summarized previous studies on the induction of CRC cell apoptosis through the interaction with gut microbes when fiber components are metabolized to butyric acid levels (Fung et al., 2012). Cheng Y et al. summarized the pathogenesis of colorectal cancer related to the microbiome. For example, Streptococcus bovis in the gut, Bacteroides flimsiformis producing enterotoxin, Escherichia coli and anaerobic streptococcus have been identified as candidate pathogens for CRC (Cheng et al., 2020). Although relevant studies have been reported in recent years (Garcia-Etxebarria et al., 2021; Ternes et al., 2022), it is not so hot now. This may be due to the shift in the focus of research on colorectal cancer from a microbiome metabolomics perspective to molecular biology to study its more specific mechanisms of occurrence (Xu et al., 2019; Garo et al., 2021; Yamada and Senda, 2021). Nonetheless, documents related to this field are still highly informative both for researchers in the field of colorectal cancer and in gastrointestinal microbiome and metabolomics.

Changes in the metabolome of the gastrointestinal microbiome have also been associated with several neurodevelopmental and neurodegenerative diseases (Grochowska et al., 2019). Moreover, diet interacts with trillions of microbiome in the gut and its metabolome and affects the organism’s metabolome directly or indirectly. It has been demonstrated that the gastrointestinal microbiome of individuals with autism differs from that of the normal population. Also, the microbiome is an instrumental part of the complex regulation of the host brain. These reports all illustrate the important impact of gastrointestinal microbiome and metabolomics on neurodegenerative diseases represented by autism spectrum disorders (Needham et al., 2018; Sanctuary et al., 2018; Pulikkan et al., 2019). Needham BD et al. used ultra-performance liquid chromatography-tandem mass spectrometry to determine metabolic molecular profiles in peripheral tissues of patients with autism spectrum disorders and revealed that the levels of phenolic microbial metabolites in the gut of altered phenolic microbial metabolites in the gut. They identified preliminary studies of metabolites that could be transferred to mice by fecal microbial transplantation (Needham et al., 2021). However, because gastrointestinal microbiome and its metabolism is affected by many factors, it is difficult to interpret these data for brain disorders such as autism spectrum disorders. Moreover, the field is still in its infancy, and much of the research is based only on animal models. Researchers need to use multiple omics techniques, a variety of omics approaches, and randomized, cross-lateral, and longitudinal studies to combine these gut microbiome and metabolomics data with information about clinical manifestations or molecular mechanisms of disorders such as autism spectrum disorders to determine whether targeting gut microbiome and metabolomics can lead to new therapeutic strategies.

In conclusion, gut microbiome metabolomics is currently at a high stage of exploration and development in relation to diseases, and the mentioned studies provide valuable insights for the treatment, diagnosis, and differential diagnosis of these diseases. Such as the search for clarification of key aspects of their interaction with the host, specific pathogenesis and the search for clinically significant biomarkers that provide independent predictive and therapeutic value for relevant clinical problems.

### 4.3 Limitations

Our study inevitably has some limitations. Initially, the original data we used were only from the WoSCC database. Despite the high comprehensiveness and reliability of the literature data from WoSCC, there may still be missing articles using the search method of a single database. Also, the articles in this study were counted only in Englisharticle and article review, making it possible to ignore some few works in different languages, as well as early access and proceeding paper. some of this may lead to biased results. Nonetheless, due to the short time interval between completing the bibliometric analysis and starting the statistics, our findings are basically not biased from the latest statistics and are informative.

## 5 Conclusion

In this study, we downloaded 3,811 original articles related to gastrointestinal microbiome and metabolomics from the WoSCC database between January 1, 2004 and December 29, 2022. And, for the first time, we used VOS viewer and CiteSpace to analyze this domain, generating various cluster analysis charts. These data-driven approaches provide a comprehensive and objective view of the current state of the field, trends, and frontiers. Through our review, statistics, and analysis, we can see that China and the United States have contributed the most to this field over the past two decades. The Journal of Proteome Research and Frontiers in Microbiology are two of the most influential journals in this field. Nicholson, Jeremy K., Jia, Wei, Holmes, Elaine and others are authoritative and important scholars in this field. Urine, spectroscopy, metabonomic, and gut microflora are long-term hot topics in this field. Studies on the specific mechanisms of small molecular metabolites and gastrointestinal microbiome in diseases have become an emerging research direction and hotspot in this field. Further study of these topics could advance the field and provide laboratory data to support the development of new treatments and outcomes for many related diseases. In conclusion, our study clarifies many important data in this field and reveals future research trends and frontiers. We hope that this study can provide relevant scholars with the information they need to increase the possibility of rapid development of the field. In addition, it also enables readers to have a more scientific, objective, and comprehensive understanding of the field.

## Data availability statement

Publicly available datasets were analyzed in this study. This data can be found here: All the data in the article could be attained from Web of Science Core Collection (WOSCC) by the search formula mentioned in the Materials and Methods.

## Author contributions

HL, LT, YW, and JW: study conception. JW, PD, and SZ: study design. PD, SZ, YW, YM, JD, and PP: study conduct. YW: Data analysis. PD, SZ, and YW: had full access to all the data in the study, taking responsibility for the integrity of the data and the accuracy of the data analysis, data interpretation, and drafting of the manuscript. PD, SZ, YW, YM, JD, and JW: critical revision of the manuscript for important intellectual content. All authors contributed to the article and approved the submitted version.
